# Investigation of the Effectiveness of Transcutaneous Auricular Vagus Nerve Stimulation (taVNS) and Vestibular Rehabilitation in Patients with Unilateral Vestibular Hypofunction

**DOI:** 10.3390/medicina61050872

**Published:** 2025-05-09

**Authors:** Tuğba Türk Kalkan, Devrim Tarakçi, Gamze Kiliç, Cengiz Çelikyurt

**Affiliations:** 1 Department of Physiotherapy and Rehabilitation, Institute of Health Sciences, Istanbul Medipol University, 34810 Istanbul, Turkey; 2 Department of Physiotherapy and Rehabilitation, Faculty of Health Sciences, Trakya University, 22180 Edirne, Turkey; 3 Department of Ergotherapy, Faculty of Health Sciences, Istanbul Medipol University, 34810 Istanbul, Turkey; 4 Department of Physiotherapy and Rehabilitation, Faculty of Health Sciences, Nisantası University, 34398 Istanbul, Turkey; 5 Clinic of Otolaryngology, Günesli Erdem Hospital, 34212 Istanbul, Turkey

**Keywords:** unilateral vestibular hypofunction, neuromodulation, transcutaneous auricular vagus nerve stimulation, vestibular rehabilitation, balance

## Abstract

*Background and Objectives*: Vagus nerve stimulation (VNS) is a frequently used neuromodulation method in recent years. While the mechanism of improvement in diseases such as epilepsy, dementia, and depression is being studied, its potential effect on vestibular dysfunction is also being investigated. The aim of our study was to investigate the effect of transcutaneous auricular VNS (taVNS) on the vestibular symptoms of unilateral vestibular hypofunction (UVH). *Materials and Methods*: Forty patients diagnosed with UVH were randomly divided into two groups. Group 1 received vestibular rehabilitation. Group 2 received taVNS and vestibular rehabilitation. Both groups received treatment one day a week for eight weeks. Before and after the treatment, balance of the participants was assessed with modified-CTSIB (m-CTSIB), limit of stability (LOS), Tandem and One-Leg Stance (OLS) tests; visual acuity was assessed with dynamic visual acuity (DVA), dizziness severity, and fatigue severity with a visual analog scale (VAS); kinesiophobia was assessed with the Tampa Scale for Kinesiophobia (TSK); depression and anxiety was assessed with the Hospital Anxiety and Depression Scale (HADS); and quality of life was assessed with the Dizziness Handicap Inventory (DHI). *Results*: At the end of eight weeks, patients in Group 2 showed significantly greater improvement in balance, dizziness, fatigue, kinesiophobia, anxiety, and depression. There was no significant difference in visual acuity and quality of life between the groups. *Conclusions*: The positive effects of taVNS on vestibular symptoms have been observed. As a new approach, taVNS can be included in the treatment of patients with UVH in addition to vestibular rehabilitation.

## 1. Introduction

Vestibular hypofunction is a clinical condition that occurs when there is a partial or complete decrease in the function of the vestibular system organs or the vestibular nerve [1]. Unilateral vestibular hypofunction (UVH) is a type of vestibular hypofunction with symptoms such as vertigo, dizziness, oscillopsia, imbalance, and gait disturbances [2]. UVH is most commonly due to vestibular neuritis but may also be due to head trauma, surgical transection, ototoxic drugs, Meniere’s disease, or other lesions of the vestibulocochlear nerve or labyrinth [2]. Treatment of vestibular hypofunction consists of vestibular rehabilitation (VR), posturography training, virtual reality, and instrumental rehabilitation [3]. In addition, antihistamines such as meclizine and vestibular suppressants such as benzodiazepines can be used in the acute treatment of vestibular hypofunction to temporarily relieve symptoms such as dizziness and nausea. However, it is known that long-term use of these drugs may inhibit central vestibular compensation. Therefore, vestibular rehabilitation is considered the main treatment approach in the long-term management of vestibular hypofunction [3].

VR is a proven and widely used treatment for patients with UVH. VR consists of patient education, a personalized structured exercise program and a home exercise program. Patient education increases the patient’s motivation and compliance with rehabilitation, allowing the home exercise program to be maintained. The vestibular exercise program includes gaze stabilization exercises, habituation exercises, exercises to improve balance and gait, and general conditioning exercises [3]. VR programs aim to facilitate adaptation of the vestibular system, improve gaze stabilization, increase dynamic visual acuity, reduce symptoms of vertigo and dizziness, alleviate movement-related vestibular symptoms, improve balance, postural control and gait stability [4,5,6]. At the same time, VR exercises facilitate return-to-normal daily activities, reduce anxiety and depression, and thus improve the quality of life of patients [7].

Vagus nerve stimulation (VNS) was first used in the treatment of drug-resistant epilepsy, and, through rigorous research, its therapeutic efficacy has been consistently confirmed [8]. The potential efficacy of invasive and non-invasive methods of VNS in the treatment of various autoimmune and chronic inflammatory disorders has attracted considerable interest. The auricular branch of the vagus nerve, which is less known than the cervical branch, has been used as a new application in the treatment of various diseases. In particular, research using transcutaneous auricular vagus nerve stimulation (taVNS), a non-invasive method that activates vagal afferent fibers, has increased [9,10].

taVNS is a non-invasive neuromodulation method. taVNS is also used as a physical therapy technique in the treatment of neurodegenerative, metabolic, inflammatory, and cardiovascular diseases and pain control [11,12,13,14,15,16,17]. taVNS’s therapeutic mechanism is hypothesized to modulate inflammatory pathways and the hypothalamic–pituitary–adrenal axis to create an anti-inflammatory environment [18,19]. Although there are no studies in the literature reporting the use of taVNS in the treatment of UVH, several recent studies have emphasized its positive effect on vestibular system diseases such as vestibular migraine [20], Meniere’s [21], benign paroxysmal positional vertigo (BPPV) [22], persistent postural-perceptual dizziness (PPPD) [23], and vestibular symptoms such as tinnitus [24], vertigo, and nystagmus [25,26,27,28]. Therefore, we suggest that taVNS can be used as a complementary treatment to vestibular rehabilitation to improve the symptoms of UVH. The aim of this study was to examine the efficacy of taVSN as a new approach in the treatment of UVH and to investigate its potential effect in combination with vestibular rehabilitation.

## 2. Materials and Methods

### 2.1. Study Design

This single-center, single blind, randomized controlled trial was conducted from 15 October 2021, to 15 June 2024 at out-patient department of otorhinolaryngology in Gunesli Erdem Hospital, Istanbul, Turkey. This study was decided to be ethically and scientifically appropriate by the Istanbul Medipol University Non-Interventional Clinical Research Ethics Committee with its decision numbered E-10840098-772.02-4966 on 4 October 2021, and it was registered at ClinicalTrials.gov NCT06332326.

Randomization was independently performed by the hospital’s institutional secretary using a computerized randomization tool (www.random.org). Interventions were delivered by a single physiotherapist who was aware of the group assignments. To minimize potential researcher bias, however, all outcome assessments were carried out by a different physiotherapist who was blinded to group allocations. Oral and written consent forms were obtained from participants.

### 2.2. Participants

This study was conducted with participants who consulted the “Otorhinolaryngology Outpatient Clinic of Istanbul/Güneşli Erdem Hospital”. Participants aged between 18 and 65 years who presented with complaints of dizziness, loss of balance, and gait disturbance and were diagnosed with UVH by videonystagmography (VNG), who had symptoms for more than 3 months from disease onset, and who had high communication skills and motivation were included in this study.

Exclusion criteria of this study were defined as lack of cooperation, having undergone any ear surgery, having central pathology findings in videonystagmography results, having central neurological disease, having pathology that prevents walking, having visual and/or cognitive impairment, conditions accompanied by acute Meniere’s disease and/or BPPV, pregnancy, and the presence of temporal bone pathologies detected by magnetic resonance imaging.

**Figure 1:**
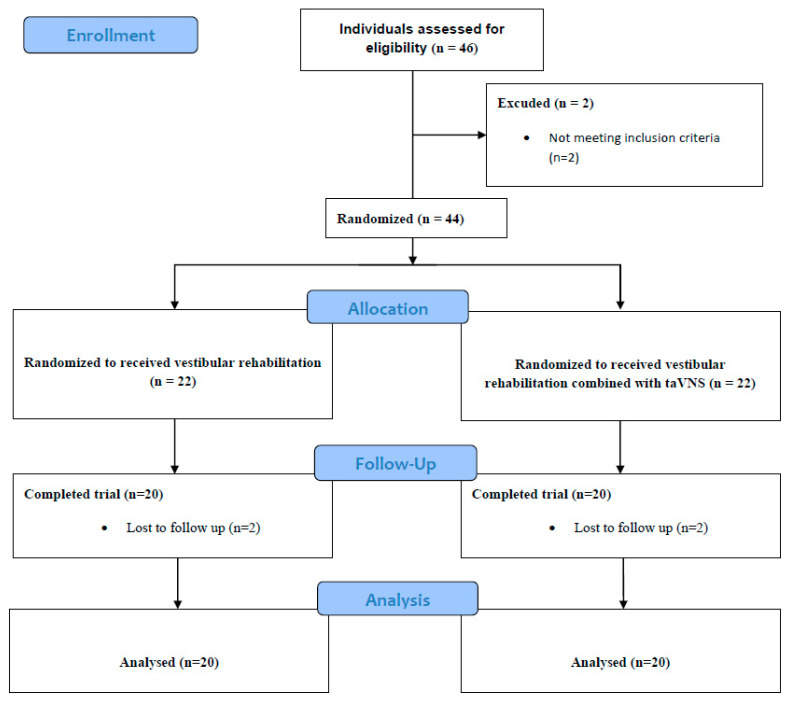
Consolidated Standards of Reporting Trials (CONSORT) flow chart for trial recruitment.

According to the inclusion and exclusion criteria, a total of 40 participants were randomly assigned to 2 separate groups. The patients in Group 1 (*n* = 20) received a vestibular rehabilitation program. The patients in Group 2 (*n* = 20) received taVNS in addition to the vestibular rehabilitation program. All participants completed the interventions and were included in the statistical analysis. The assignment of the patients to the study groups is shown in Figure 1.

G*Power 3.1.9.7 was used to determine the sample size of this study. The effect size was 0.95, the significance level was 0.05, the power value was 0.80, and the intergroup distribution ratio was 1. As a result of the analysis, the number of participants in the groups was determined as 19. Participants were informed about the purpose of this study, evaluations, and treatment programs. A signed informed consent form was obtained from the participants indicating that they voluntarily participated in this study.

The initial evaluation of the patients was performed by an otolaryngologist. These evaluations consisted of physical examination and VNG test. Patients were then referred to a vestibular physiotherapist for vestibular physiotherapy evaluation and treatment.

### 2.3. Outcome Measurements

After demographic information was obtained, the participants were administered; modified-CTSIB (m-CTSIB) and limits of stability (LOS) tests for objective assessment of postural control and balance [29]; Tandem and One-Leg Stance (OLS) tests for static balance assessment; dynamic visual acuity (DVA) test for visual acuity; visual analog scale (VAS) for dizziness severity and fatigue severity; Dizziness Disability Inventory (DHI) for quality of life; Tampa Scale for Kinesiophobia (TSK) for kinesiophobia; and Hospital Anxiety and Depression Scale (HADS) for depression and anxiety. All participants were evaluated twice: before and after eight weeks of treatment.

#### 2.3.1. Demographic Information Form

A structured form was prepared to collect sociodemographic characteristics of the participants such as gender, age, smoking habits, alcohol consumption (Table 1). The accompanying comorbidities and medical history of the patients in both groups are presented in Supplementary Table S1.

#### 2.3.2. LOS

The LOS test was performed using a posturography device (ICS Balance Platform, GN Otometrics, Taastrup, Denmark), which includes a computerized force platform system to objectively assess dynamic balance. With this system, body movements are detected according to the pressure of the person’s foot on the force platform. In this test, the participant, with arms by the side of the body, with feet spread about 30° apart, focuses straight ahead and responds to instructions in 8 directions (Forward, Right Forward, Right, Right Back, Right Back, Back, Left Back Left, Left Forward) randomly displayed on a monitor at a distance of 50 cm by shifting the center of mass as far as possible in the corresponding direction once and then returning to the center, with feet remaining stationary on the ground. Reaction time (RT), movement velocity (MVL), end point reached (EP), maximum excursion (MXE), and directional control (DC) sections of the test, which are displayed in eight directions, increase its reliability [29,30].

#### 2.3.3. m-CTSIB

Modified clinical test of sensory integration in balance (mCTSIB) test, was performed with a posturography device (ICS Balance Platform, GN Otometrics, Taastrup, Denmark) including a computerized force platform system to objectively assess postural stability and balance. mCTSIB test assesses postural stability in 4 conditions: standing on a hard surface with eyes open, then with eyes closed; standing on soft surface (medium density square 40 × 40 × 15 cm) with eyes open and then with eyes closed. Postural stability was observed in each condition, and body oscillations (mm/s) were recorded by the device [29].

#### 2.3.4. Static Balance Tests

Tandem and OLS (One-Leg Stance) tests were used to evaluate balance. In the Tandem and OLS tests, participants were asked to stand on hard and soft surfaces with their eyes open and closed for 30 s. Balance times (seconds) were recorded [31].

#### 2.3.5. DVA

The patient’s head was passively moved rhythmically in the horizontal plane with an amplitude of 20° and a speed of 2 Hz. Meanwhile, the patient was asked to start reading the letters on the Snellen card. The clearly visible line was recorded [32].

#### 2.3.6. VAS

Dizziness and fatigue severity (VAS) were assessed. A 10 cm visual analog scale was used for the assessment. Patients were asked to rate the severity of dizziness between 0 and 10. It was explained that a score of “0” indicates no dizziness and a score of “10” indicates the presence of unbearable dizziness, and the patient was asked to mark the appropriate part [29].

#### 2.3.7. TSK

This was used to measure fear of movement and fear of re-injury. The scale consists of 17 questions with 4-point Likert scoring (1 = strongly disagree, 4 = strongly agree). Items 4, 8, 12, and 16 were reversed, and the total score is calculated. The total score takes a value between 17 and 68. A high score indicates high kinesiophobia [33].

#### 2.3.8. HADS

This was used to assess the level of depression and anxiety. The scale has a total of 14 items. Seven of the scale items determines the level of anxiety, and seven determines the level of depression [23].

#### 2.3.9. DHI

This was used to assess the impact of dizziness on quality of life. The DHI consists of 3 sub-dimensions and a total of 25 items aiming to determine the physical, emotional, and functional effects of vestibular system diseases [34].

### 2.4. Treatment Programs

After initial evaluations, the patients included in this study were randomly divided into two groups. Patients in Group 1 received a vestibular rehabilitation program. Patients in group 2 received taVNS in addition to the vestibular rehabilitation program. Participants were not administered any pharmacological treatment during the intervention period.

#### 2.4.1. Vestibular Rehabilitation Group (Group 1, *n* = 20)

The vestibular rehabilitation program was developed based on the clinical practice guideline and evidence from the current literature [29,33,35]. The program included vestibular adaptation exercises, oculomotor exercises, static and dynamic balance exercises, posture exercises, and walking exercises. Each participant’s program was individually tailored and delivered by a vestibular physiotherapist once per week in 45–50 min sessions for 8 weeks. Exercises were progressed from simple to complex to minimize side effects such as dizziness, nausea, or falls. Patients were also prescribed a home exercise program with 10 repetitions, three times daily. Patient education and instructional materials (illustrated leaflets and verbal explanations) were provided to support home compliance. All participants performed the full set of exercises during each supervised session under the guidance of the same physiotherapist, and weekly progressions were standardized. Compliance with the home exercise program was monitored verbally at each follow-up. All patients were able to perform all prescribed exercises in each supervised session and continued the home exercise program regularly. The details of the exercise program are presented in Table 2.

#### 2.4.2. Vestibular Rehabilitation Group Combined with taVNS (Group 2, *n* = 20)

**Figure 2:**
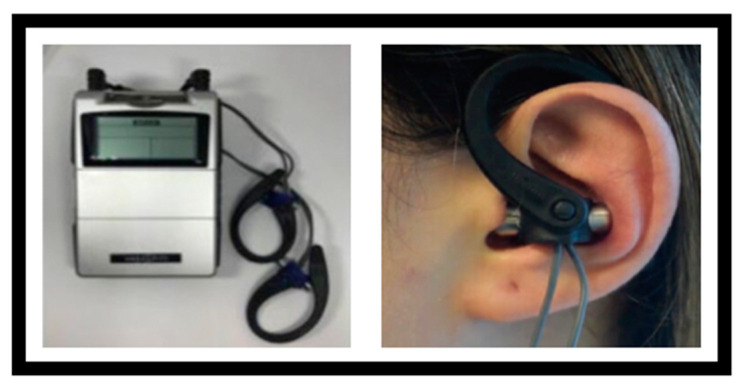
Transcutaneous auricular vagus nerve stimulation (taVNS) application.

Participants in Group 2 received the same vestibular rehabilitation program as described for Group 1, including both supervised sessions and the home exercise program. In addition to this full vestibular rehabilitation protocol, taVNS therapy was administered using the Vagustim^®^ device (Vagustim Health Technologies Inc., Istanbul, Turkey). Stimulation was delivered through specially designed headphones with electrodes adapted to the size of the ear canal. The electrodes were bilaterally placed on the tragus and concha regions, targeting the auricular branch of the vagus nerve (Figure 2). Bilateral application was preferred to maximize neuromodulatory effects [36]. The stimulation parameters were set at a frequency of 10 Hz and a pulse duration of 300 µs, delivered in TENS mode using a biphasic asymmetric waveform [37]. The current intensity was gradually increased until the participant reported a comfortable sensation and ranged between 0.13 and 50 mA above threshold. If discomfort or pain occurred, the intensity was adjusted accordingly. The stimulation was applied for 15 min, once per week, following the vestibular rehabilitation session, over a period of 8 consecutive weeks. All participants in Group 2 completed the same vestibular rehabilitation program as in Group 1, including the supervised and home-based exercises, without missing any components. Additionally, all participants received the taVNS intervention once per week for eight consecutive weeks, in accordance with the protocol.

### 2.5. Statistical Analysis

The data obtained in this study were analyzed using SPSS 18 (Statistical Package for Social Sciences). Number, percentage, mean, standard deviation, minimum, and maximum values were used as descriptive statistical methods in the evaluation of the data. The assumption of normality was tested before comparing within and between group differences. Since the number of observations was less than 50, Shapiro–Wilk values were taken into consideration. In intra-group difference comparisons, when the assumptions were met, the dependent sample *t* test was applied from parametric tests, and, when the assumptions were not met, the Wilcoxon test was applied from nonparametric tests. In between-group difference comparisons, independent sample t test, one of the parametric tests, and Mann–Whitney u test, one of the nonparametric tests, were applied. In this study, *p* < 0.05 was considered statistically significant.

## 3. Results

A total of 46 patients were included in this study. Two of the patients did not meet the inclusion criteria. A total of 44 people were randomized into two separate groups, 22 in each group. Four patients did not complete this study. As shown in the patient flow chart (Figure 1), 40 people were analyzed (group 1, *n* = 20 and group 2, *n* = 20). Demographic data of the patients are shown in Table 1.

According to the results in LOS parameters, all patients showed improvement. However; more significant improvement was seen in LOS parameters of patients in Group 2 (*p* < 0.01). According to m-CTSIB results; patients in Group 2 showed significant improvement (*p* < 0.01), while the improvement in Group 1 was not statistically significant. LOS and m-CTSIB results are shown in Table 3.

According to VAS, TSK, and HADS results shown in Table 4, significant improvements were seen in all patients. However, when comparing between the groups, patients in Group 2 showed significantly more improvement than patients in Group 1 (*p* < 0.01), (*p* < 0.05). According to Tandem, OLS, DVA, DHI results shown in Table 4, all patients improved significantly (*p* < 0.01), (*p* < 0.05), but there was no significant difference between the groups.

## 4. Discussion

In our study investigating the effect of taVNS in patients with UVH, balance, dizziness, fatigue, kinesiophobia, depression, and anxiety improved significantly more in patients who received taVNS in addition to vestibular rehabilitation compared to patients who received only vestibular rehabilitation.

VNS is emerging as a potential new approach in various musculoskeletal disorders [38]. VNS affects numerous physiological processes involving brain–body interaction through modulation of the afferent branch of the vagus nerve. The superficial branch of the vagus nerve extending to the auricle creates a site for cutaneous stimulation in the external ear [14]. Nowadays, convenient taVNS devices that allow noninvasive application offer a safe treatment option. In a few studies examining the effect of taVNS on vestibular system diseases and symptoms, positive effects have been reported [20,21,22,23]. However, in the current literature, there is no study examining the effect of taVNS in the treatment of UVH. With our study, we would like to draw attention to the positive effect of taVNS on balance, dizziness, fatigue severity, and depression level.

In the literature, the pathomechanisms underlying the positive effect of VNS on postural control and balance through autonomic nervous system modulation are not well understood. In one study, vertigo severity and postural oscillation were significantly reduced with VNS administration [23]. Similarly, in our study, taVNS was found to reduce vertigo severity and improve postural control and balance in patients with UVH. In our study, we found that patients who received taVNS in addition to vestibular rehabilitation improved significantly more than patients who did not receive taVNS. We think that this difference is due to the effect of taVNS on the parasympathetic nervous system and cortical areas. taVNS not only stimulates the vagus nerve, which is an important part of the parasympathetic nervous system through neuromodulation, but can also stimulate the central regions of this nerve, including cortical areas [39,40,41,42,43]. There is evidence that lesions of the insular cortex can lead to dizziness and autonomic dysfunctions, while the cingulate cortex plays a critical role in depression and anxiety [39,44,45,46,47]. Stimulation of these areas may occur with the release of norepinephrine after taVNS. The greater improvement of postural control and balance in patients with taVNS may be explained by the hypothesized important effects of the insular and cingulate cortex on postural control.

Studies have reported that patients with unilateral vestibular hypofunction experience negative postural control and loss of balance. Vestibular rehabilitation is known to reduce body oscillations and improve balance capacity [48,49]. In existing studies, the improvement of postural control and balance after vestibular rehabilitation has been investigated with different assessment methods. In our study, both timed clinical balance tests such as Tandem and One-Leg Stand tests and objective assessment methods such as m-CTSIB and LOS with computerized posturography device were used. With the evaluation tests we used in our study, it was aimed to reveal the effect of vestibular rehabilitation and taVNS in detail and to fill the lack of the literature on the effect of taVNS on balance tests. Studies evaluating body oscillations and balance with LOS and m-CTSIB have found significant improvements after vestibular rehabilitation [30]. In our study, postural control and balance improved in all patients after vestibular rehabilitation, consistent with previous studies. Moreover, statistically more significant improvement was observed in patients who underwent taVNS compared to patients in the control group. However, this difference was not observed in Tandem and OLS tests. We think that the effect of taVNS, which we applied as a complement to vestibular rehabilitation, on balance can be demonstrated with more objective and sensitive assessment tests such as m-CTSIB, and LOS instead of timed balance tests.

In the pathophysiology of vestibular hypofunction, there is a decrease in the tone of the vestibular nuclei and vestibulo-ocular reflex (VOR). When the VOR response decreases, dynamic visual acuity is impaired. In studies, visual acuity increased, and dizziness decreased as a result of a vestibular exercise program based on head and eye movements [50]. In our study, VOR exercises were included in the structured vestibular program because of their positive effects on VOR response, dynamic visual acuity, and dizziness. The 8-week structured vestibular rehabilitation program improved the vestibulo-ocular reflex and restored dynamic visual acuity in our patients. As a result of our study, according to the DVA test, dynamic visual acuity improved significantly in patients with UVH. There were also significant improvements in dizziness and fatigue. We think that especially oculomotor and vestibular adaptation exercises have a great effect on the significant improvement in dizziness and fatigue.

Studies examining the effect of vestibular rehabilitation on fatigue have generally been conducted on individuals with neurological diseases. It is reported that vestibular rehabilitation program reduces fatigue and increases activities of daily living in individuals with neurological diseases such as MS, Parkinson’s, and stroke [51,52,53]. In 2023, Genc et al. evaluated fatigue in patients with bilateral vestibular hypofunction (BVH) after a 12-week individualized structured vestibular rehabilitation program. In this study, which provides the first evidence on the subject, it was observed that fatigue decreased, although not significantly [33]. Along with this study, our study also evaluated fatigue levels in UVH, one of the types of vestibular hypofunction. However, in the results of our study, fatigue was significantly reduced. In addition, fatigue decreased significantly more in patients undergoing taVNS compared to patients undergoing isolated vestibular rehabilitation. We think that the autonomic nervous system effect of taVNS reduces fatigue more, and further studies on this subject are needed.

In recent years, studies have been conducted on kinesiophobia triggered by dizziness. In a study conducted in patients with UVH, an 8-week vestibular rehabilitation program significantly reduced TSK scores in kinesiophobia evaluations [54]. In our study, vestibular rehabilitation significantly reduced kinesiophobia in patients with UVH according to TSK scores. In addition to the contributions of improved dizziness and balance, we think that kinesiophobia was significantly improved with various dynamic-static balance exercises and walking exercises in the structured vestibular program that we applied in our patients. At the same time, with the supervised and repetitive application of the exercises, we think that the patients overcame their fear of movement due to the fear of dizziness and imbalance. In addition, our study found that the combination of taVNS and vestibular rehabilitation was significantly superior in reducing kinesiophobia. This superiority may be explained by the hypothesis that taVSN contributes to the improvement of kinesiophobia in a manner similar to the vagus–autonomic nervous system–depression relationship, in addition to the conclusion that additional taVSN improves dizziness and balance more.

In the literature, sustained activation of the autonomic nervous system has been discussed as an important pathophysiological pathway in the development of depression, and the effect of VNS on the treatment of depression has been proven by many studies [55]. On the other hand, it is known that the quality of life of patients with vestibular hypofunction is adversely affected, and depression and anxiety levels increase due to the symptoms that occur in individuals with vestibular hypofunction. In addition to significantly reducing vertigo severity and postural oscillation, studies have reported the positive effects of VNS on psychiatric comorbidities such as depression and anxiety [21,22,23]. In our study, which we designed considering the possible positive effects of taVNS on depression and anxiety levels in patients with UVH, significant differences were found according to HADS scores. Patients who received taVNS in addition to vestibular rehabilitation had significantly improved levels of depression and anxiety compared to patients who received vestibular rehabilitation alone. The results of our study suggest that taVSN has an indirect effect on depression through its positive effects on dizziness severity and balance and a direct effect through autonomic nervous system modulation.

VR is known to improve quality of life in patients with vestibular hypofunction by reducing dizziness and loss of balance [54]. In existing studies, the quality of life of patients with vestibular dysfunction has typically been assessed using the DHI. The DHI has been shown to correlate with the Short Form-36 Health Survey (SF-36), a widely used instrument for evaluating health-related quality of life across multiple domains [56]. Therefore, in our study, quality of life in patients with UVH was also assessed by the DHI, and quality of life was significantly improved in all patients. We suggest that an 8-week structured vestibular rehabilitation program improves dizziness and balance, thus improving kinesiophobia, depressive symptoms, and quality of life.

In comparison with the randomized controlled trial conducted by Wu et al. (2023), which evaluated the effects of taVNS combined with Betahistine Mesylate in individuals with Meniere’s disease, our study offers a novel contribution by investigating the standalone neuromodulatory role of taVNS when integrated with vestibular rehabilitation in patients with UVH [21]. While Wu et al. demonstrated clinical improvements in hearing thresholds, tinnitus, aural fullness, and overall quality of life—largely attributed to the synergistic effect of pharmacologic and neuromodulatory interventions—our study eliminated the potential confounding influence of medication and isolated the therapeutic contribution of taVNS. Furthermore, the present investigation employed a broader array of outcome measures, including kinesiophobia, fatigue, anxiety, and depression. These methodological distinctions not only underscore the originality of our work but also extend the evidence base supporting taVNS as a promising adjunct in the treatment of vestibular dysfunctions beyond Meniere’s disease, particularly in non-pharmacologic rehabilitation settings.

In our study, it was observed that patients diagnosed with unilateral vestibular hypofunction had significant improvements in balance parameters after vestibular rehabilitation. It was also found that there was a decrease in the severity of dizziness and thus improvement in kinesiophobia scores. In addition, it was determined that vestibular exercises increased VOR response and gaze stabilization, decreased fatigue, anxiety, and depression levels, and increased the quality of life of the patients. Our findings are consistent with the existing literature and contribute to the literature by showing that the integration of taVNS into vestibular rehabilitation provides greater improvement in vestibular symptoms in individuals with unilateral vestibular hypofunction.

## 5. Conclusions

In conclusion, taVNS treatment in addition to vestibular rehabilitation improved dizziness, balance, fatigue, kinesiophobia, anxiety, and depression levels more than vestibular rehabilitation treatment in isolation. Our study conclusively demonstrates the positive effect of taVNS treatment on balance and depression levels in patients with UVH.

As a new approach, taVNS can be added to rehabilitation strategies to treat vestibular dysfunction.

### Limitations

The lack of a placebo or sham stimulation group makes it difficult to attribute the observed effects to vagus stimulation treatment. In addition, the lack of long-term follow-up data limits the assessment of treatment durability.

## Figures and Tables

**Table 1:** Demographic data description.

		Group 1 (*n* = 20)	Group 2 (*n* = 20)
**Gender (*n*/%)**	*Female*	16/80	7/35
	*Male*	4/20	13/65
**Age (mean ± SD)**		46.40 ± 13.28	45.00 ± 13.21
**Cigarettes (*n*/%)**	*Yes*	4/20	10/50
	*No*	16/80	10/50
**Alcohol (*n*/%)**	*Yes*	0	2/10
	*No*	20/100	18/90

Mean = mean, SD = standard deviation.

**Table 2:** Vestibular rehabilitation program applied to both groups.

	Definition	Exercises
**Vestibular adaptation exercises**	VOR*1 VOR*2 The VOR response is developed by activating the adaptation mechanism. Head movements are made during visual fixation. Includes saccadic and pursuit eye movements.	- Moving the head to the right/left/up/down in a sitting position with gaze fixed on the target - Moving the head to the right/left/up/down on hard and soft ground, feet together/tandem/semitandem positions with gaze fixed on the target - Turning the head to the right/left in the direction opposite to the movement while the eyes are fixed on the object moving to the right/left while sitting/standing
**Oculomotor exercises**	Develops the visual system. Includes saccadic and pursuit eye movements.	- Visual tracking of an object moving to the right/left/up/down while sitting/standing with head still
**Static and dynamic balance exercises**	This includes balance exercises in which the support area, support surface, arm position, head and eye movements are varied.	- Bending forward with the ball while sitting/standing - Reaching forward while sitting/standing - Standing on one leg with eyes open/closed on hard/soft floor - Catching the ball thrown from different directions in the stance position on hard/soft floor - Throwing the ball in different directions in stance position on hard/soft floor
**Posture exercises**	This includes exercises especially for the neck, shoulder, and back muscles.	- Neck exercises with eyes open/closed while sitting/standing - Neck exercises with eyes open/closed on hard and soft floor with feet together/tandem/semitandem positions - Shoulder mobilization - Strengthening the back extensors
**Walking exercises**	It includes walking and balance exercises with different tasks.	- Normal walking on hard/soft floor with eyes open/closed - Eyes open/closed, soldier’s gait/walking backwards/tandem walking/walking in a line - Eyes open/closed, hands at side/back/shoulder crossed normal/tandem/backwards gait - Looking around, hands at side/back/shoulder crossed normal/tandem/backward gait - During walking exercises, stopping suddenly, changing direction, and maintaining various cognitive activities

VOR = Vestibulo-ocular reflex.

**Table 3:** Intra-group and inter-group comparison of objective assessment of postural control and balance.

			Group 1			Group 2			*p*
			Before Treatment	After Treatment	*p*	Before Treatment	After Treatment	*p*	
**LOS**	*MV (°/s)*	*Forward*	4.55 ± 0.8	4.59 ± 0.55	0.883	4.66 ± 0.59	4.91 ± 0.75	0.250	0.260
		*Right Forward*	4.42 ± 0.8	4.71 ± 0.89	0.290	4.97 ± 0.84	5.04 ± 0.68	0.646	0.788
		*Right*	3.67 ± 0.87	3.94 ± 0.58	0.166	3.92 ± 0.59	4.07 ± 1.04	0.444	0.255
		*Right Backward*	2 ± 0.51	2.16 ± 0.81	0.585	2.14 ± 0.73	2.31 ± 0.83	0.513	0.684
		*Backward*	1.1 ± 0.32	1.16 ± 0.26	0.739	1.14 ± 0.35	1.21 ± 0.35	0.581	0.488
		*Left Backward*	1.99 ± 0.46	2.11 ± 0.51	0.475	2.4 ± 0.51	2.29 ± 0.71	0.485	0.946
		*Left*	3.59 ± 0.66	3.7 ± 0.8	0.626	4.01 ± 0.59	4.18 ± 0.63	0.350	0.178
		*Left Forward*	4.76 ± 0.83	4.99 ± 0.87	0.266	4.95 ± 0.72	5.43 ± 0.91	0.020 *	0.068
	*EP (%)*	*Forward*	70.45 ± 19.7	73 ± 11.75	0.659	77.15 ± 12.11	84.05 ± 10.17	0.081	0.059
		*Right Forward*	77.8 ± 16.87	81.95 ± 17.42	0.433	88.2 ± 19.56	90.05 ± 14.17	0.590	0.734
		*Right*	72.4 ± 18.99	77.25 ± 12.53	0.309	76.75 ± 14.92	80.8 ± 9.34	0.227	0.311
		*Right Backward*	69.25 ± 25.2	79.55 ± 18.79	0.088	73.45 ± 18.79	79 ± 17.29	0.259	0.337
		*Backward*	70.95 ± 20.12	79.9 ± 18.51	0.088	68.5 ± 21.41	76.4 ± 20.66	0.191	0.242
		*Left Backward*	71.85 ± 21.22	79.3 ± 17.71	0.238	80.4 ± 14.38	79.05 ± 13.74	0.743	0.763
		*Left*	72.5 ± 13.96	76.1 ± 14.4	0.446	79.2 ± 9.33	80.35 ± 11.71	0.709	0.733
		*Left Forward*	86.2 ± 20.22	89.9 ± 19.58	0.409	89.1 ± 16.56	93.75 ± 16.79	0.160	0.383
	*MXE (%)*	*Forward*	79.45 ± 15.46	80.7 ± 11.31	0.755	82.95 ± 13.25	89.75 ± 7.4	0.070	0.054
		*Right Forward*	87.45 ± 9.96	88.65 ± 11.59	0.684	93.65 ± 17.29	94.8 ± 12.35	0.779	0.735
		*Right*	80.05 ± 15.04	81.45 ± 8.96	0.709	82.55 ± 10.46	82.65 ± 7.75	0.965	0.973
		*Right Backward*	74.4 ± 22.7	83.8 ± 17.88	0.032 *	82.9 ± 12.23	90.75 ± 9.84	0.004 **	0.031 *
		*Backward*	86.45 ± 32.74	85.6 ± 13.82	0.727	79.4 ± 18.5	86.9 ± 14.8	0.097	0.165
		*Left Backward*	77.1 ± 16.87	84.5 ± 14.76	0.154	86.6 ± 8.85	87.1 ± 10.08	0.801	0.868
		*Left*	80.6 ± 12.12	126.95 ± 195.11	0.239	81.65 ± 8.68	84.1 ± 8.33	0.275	0.368
		*Left Forward*	91 ± 17.86	95.75 ± 15.23	0.303	93.65 ± 13.19	97.9 ± 11.57	0.098	0.286
	*RT (s)*	*Forward*	1.25 ± 0.3	1.17 ± 0.36	0.147	1.18 ± 0.49	0.92 ± 0.41	0.044 *	0.317
		*Right Forward*	1.01 ± 0.39	0.98 ± 0.23	0.501	0.99 ± 0.25	0.92 ± 0.27	0.286	0.267
		*Right*	0.95 ± 0.22	0.88 ± 0.27	0.881	0.95 ± 0.18	0.93 ± 0.16	0.709	0.946
		*Right Backward*	1.2 ± 0.32	0.95 ± 0.33	0.014 *	0.86 ± 0.42	0.88 ± 0.17	0.616	0.329
		*Backward*	1.04 ± 0.24	0.96 ± 0.26	0.376	0.95 ± 0.23	0.9 ± 0.11	0.267	0.154
		*Left Backward*	1 ± 0.27	0.86 ± 0.26	0.079	0.99 ± 0.2	0.9 ± 0.2	0.014 *	0.072
		*Left*	0.95 ± 0.35	0.94 ± 0.15	0.629	0.88 ± 0.27	0.87 ± 0.19	0.055	0.227
		*Left Forward*	1.43 ± 2.13	0.96 ± 0.11	0.324	1.01 ± 0.33	0.99 ± 0.16	0.871	0.875
	*DC (%)*	*Forward*	83.35 ± 12	79.8 ± 10.07	0.165	81.1 ± 10.49	79.95 ± 13.84	0.837	0.914
		*Right Forward*	68.55 ± 14.04	67.95 ± 16.87	0.914	73.85 ± 11.61	73 ± 13.47	0.629	0.989
		*Right*	69.6 ± 11.35	70.75 ± 14.6	0.499	76.9 ± 12.74	76.6 ± 9.39	0.935	0.933
		*Right Backward*	64.2 ± 14.41	63.6 ± 15.83	0.908	62.15 ± 19.05	67.3 ± 14.02	0.422	0.310
		*Backward*	62.95 ± 23.87	72.55 ± 19.59	0.260	76 ± 16.43	70.2 ± 16.95	0.324	0.228
		*Left Backward*	68.25 ± 15.78	67.25 ± 14.92	0.823	69.05 ± 14.28	67.85 ± 14.85	0.867	0.560
		*Left*	74.7 ± 17.81	76.5 ± 12.32	0.763	70.25 ± 14.52	71.6 ± 13.88	0.735	0.765
		*Left Forward*	70.6 ± 17.15	73.3 ± 12.24	0.643	74.25 ± 10.71	72.7 ± 11.56	0.685	0.662
**m-CTSIB** **(mm/s)**	*Hard*	*EO*	9.47 ± 2.17	8.93 ± 2	0.161	11.09 ± 3.2	9.48 ± 2.01	0.045 *	0.065
		*EC*	13.55 ± 5.59	12.22 ± 4.11	0.205	14.63 ± 5.55	11.39 ± 4.06	0.004 **	0.016 *
	*Soft*	*EO*	12.95 ± 3.37	11.94 ± 3.07	0.062	12.29 ± 2.34	11.85 ± 2	0.372	0.532
		*EC*	21.38 ± 8.5	18.74 ± 7.4	0.179	22.27 ± 8.72	16.28 ± 5.01	0.002 **	0.019 *

LOS = limit of stability; MV = movement velocity; EP = endpoint; MXE = maximum excursion; RT = reaction time; DC = directional control; Hard = hard floor; Soft = soft floor; EO = eyes open; EC = eyes closed °/s = degrees/second; s = seconds; % = percent; mm/s: millimeter/second. Intragroup comparison, summary statistics are given as mean ± standard value. * Significant outcome (*p* < 0.05); ** Highly significant outcome (*p* < 0.01).

**Table 4:** Intra-group and inter-group comparison of other outcome measures.

			Group 1			Group 2			*p*
			Before Treatment	After Treatment	*p*	Before Treatment	After Treatment	*p*	
**Tandem (s)**	*EO*		28.73 ± 3.07	30 ± 0	0.068	26.7 ± 8.11	30 ± 0	0.109	
	*EC*		14.27 ± 8.25	26.13 ± 5.92	0.000 **	16.85 ± 13.28	27.96 ± 4.7	0.003 **	0.377
**OLS (s)**	*Hard EO*	*Right foot*	23.64 ± 9.54	28.41 ± 5.07	0.008 **	25.98 ± 9.24	29.18 ± 2.73	0.068	0.917
		*Left foot*	25.84 ± 9.03	28.56 ± 4.45	0.043 *	23.8 ± 10.49	28.65 ± 3.28	0.028 *	0.486
	*Hard EC*	*Right foot*	9.14 ± 8.69	20.44 ± 9.8	0.000 **	10.06 ± 8.32	25.42 ± 7.75	0.000 **	0.101
		*Left foot*	8.28 ± 7.56	19.24 ± 10.32	0.000 **	8.81 ± 7.74	23.05 ± 8.7	0.000 **	0.216
	*Soft EO*	*Right foot*	19.86 ± 11.37	27.12 ± 5.61	0.003 **	25.12 ± 7.75	28.21 ± 4.24	0.018 *	0.579
		*Left foot*	19.2 ± 12.49	26.95 ± 6.41	0.004 **	21.74 ± 9.84	28.4 ± 4.24	0.003 **	0.283
	*Soft EC*	*Right foot*	5.78 ± 6.57	15.39 ± 9.92	0.000 **	5.85 ± 4.32	20.1 ± 8.57	0.000 **	0.079
		*Left foot*	6.12 ± 7.71	15.23 ± 10.28	0.000 **	4.76 ± 3.4	18.06 ± 8.06	0.000 **	0.242
**DVA**			0.35 ± 0.34	0.15 ± 0.26	0.003 **	0.53 ± 0.35	0.32 ± 0.4	0.013 *	0.146
**Dizziness**	*VAS*		7.33 ± 1.33	1.57 ± 1.08	0.000 **	6.24 ± 1.9	0.18 ± 0.46	0.000 **	0.000 **
**Fatigue**	*VAS*		7.38 ± 1.67	2.8 ± 1.3	0.000 **	7.36 ± 2.18	1.09 ± 1.3	0.000 **	0.000 **
**TSK**			50.2 ± 3.61	27.2 ± 1.74	0.000 **	49.3 ± 3.74	24.35 ± 1.73	0.000 **	0.000 **
**HADS**			21.2 ± 5.18	13.1 ± 5.1	0.000 **	21.15 ± 5.86	7.9 ± 4.02	0.000 **	0.003 **
**HADS-A**			11.9 ± 2.55	7.3 ± 2.6	0.000 **	11.8 ± 2.76	4.6 ± 2.37	0.000 **	0.001 **
**HADS-D**			9.3 ± 3.06	5.8 ± 2.78	0.000 **	9.35 ± 3.5	3.3 ± 2.15	0.000 **	0.004 **
**DHI**			58.4 ± 25.48	5.5 ± 7.37	0.000 **	82.1 ± 19.63	6 ± 6.62	0.000 **	0.761

OLS = One-Leg Stance test; DVA = dynamic visual acuity; VAS = visual analog scale; TSK = Tampa Scale for Kinesiophobia; HADS = Hospital Anxiety and Depression Scale; HADS-A = Hospital Anxiety and Depression Scale-Anxiety; HADS-D = Hospital Anxiety and Depression Scale-Depression; DHI = Dizziness Handicap Inventory; Hard = hard floor; Soft = soft floor; EO = eyes open; EC = eyes closed; s = seconds. Intragroup comparison, summary statistics are given as mean ± standard value. * Significant outcome (*p* < 0.05); ** Highly significant outcome (*p* < 0.01).

## Data Availability

The datasets generated during and/or analyzed during the current study are not publicly available, but they are available from the corresponding author upon reasonable request.

## Supplementary Materials

The following supporting information can be downloaded at https://www.mdpi.com/article/10.3390/medicina61050872/s1, Table S1: Comorbidities and medical history of the patients.

## Abbreviations

taVNS: transcutaneous auricular vagus nerve stimulation; VNS: vagus nerve stimulation; UVH: unilateral vestibular hypofunction; m-CTSIB: Modified Clinical Test of Sensory Integration in Balance; LOS: limit of stability, OLS: One-Leg Stance, DVA: dynamic visual acuity, VAS: visual analog scale, TSK: Tampa Scale for Kinesiophobia, HADS: Hospital Anxiety and Depression Scale, DHI: Dizziness Handicap Inventory, VR: vestibular rehabilitation, and VOR: vestibulo-ocular reflex.
