# Supplementary material for: Investigation of the Effectiveness of Transcutaneous Auricular Vagus Nerve Stimulation (taVNS) and Vestibular Rehabilitation in Patients with Unilateral Vestibular Hypofunction
Source: Medicina (Kaunas). 2025 May 9;61(5):872. doi: 10.3390/medicina61050872 (PMC12113213; doi:10.3390/medicina61050872)
Supplement: Supplementary file 1 [file medicina-61-00872-s001.zip › medicina-3571438-supplementary.pdf]

Supplementary Table S1. Comorbidities and medical history of the patients.

Group	Participant ID	Comorbidities-Medical History
Group 1	G1-P01	Hyperlipidemia
Group 1	G1-P02	Musculoskeletal disorders-COVID-19 history
Group 1	G1-P03	
Group 1	G1-P04	Hyperlipidemia
Group 1	G1-P05	Musculoskeletal disorders-Recurrent upper respiratory tract infections history
Group 1	G1-P06	Endocrine and metabolic disorders
Group 1	G1-P07	Endocrine and metabolic disorders-Pneumonia history
Group 1	G1-P08	Musculoskeletal disorders
Group 1	G1-P09	Musculoskeletal disorders
Group 1	G1-P10	Musculoskeletal disorders- COVID-19 history
Group 1	G1-P11	Hypertension, Diabetes mellitus
Group 1	G1-P12	
Group 1	G1-P13	Diabetes mellitus-Pneumonia history
Group 1	G1-P14	Endocrine and metabolic disorders
Group 1	G1-P15	Hypertension, Diabetes mellitus
Group 1	G1-P16	Hypertension,
Group 1	G1-P17	Diabetes mellitus
Group 1	G1-P18	Endocrine and metabolic disorders-Pneumonia history
Group 1	G1-P19	COVID-19 history
Group 1	G1-P20	COVID-19 history
Group 2	G2-P01	Endocrine and metabolic disorders
Group 2	G2-P02	Hyperlipidemia
Group 2	G2-P03	Musculoskeletal disorders-Recurrent upper respiratory tract infections history
Group 2	G2-P04	Hypertension
Group 2	G2-P05	Hypertension, Diabetes mellitus
Group 2	G2-P06	Hypertension- Recurrent upper respiratory tract infections history
Group 2	G2-P07	
Group 2	G2-P08	COVID-19 history
Group 2	G2-P09	
Group 2	G2-P10	Hypertension, Diabetes mellitus
Group 2	G2-P11	Hyperlipidemia
Group 2	G2-P12	Endocrine and metabolic disorders
Group 2	G2-P13	COVID-19 history
Group 2	G2-P14	
Group 2	G2-P15	Hyperlipidemia-Pneumonia history
Group 2	G2-P16	Diabetes mellitus
Group 2	G2-P17	Diabetes mellitus- COVID-19 history
Group 2	G2-P18	COVID-19 history
Group 2	G2-P19	COVID-19 history
Group 2	G2-P20	Diabetes mellitus
